# Transcription factor CEBPB mediates intracranial aneurysm rupture by inflammatory and immune response

**DOI:** 10.1111/cns.14603

**Published:** 2024-02-08

**Authors:** Zhongbin Tian, Xuefang Wu, Baorui Zhang, Wei Li, Chao Wang

**Affiliations:** ^1^ Department of Interventional Radiology, Beijing Friendship Hospital Capital Medical University Beijing China; ^2^ Department of Oncology The First Affiliated Hospital of Nanjing Medical University Nanjing China; ^3^ Department of Neurosurgery, Beijing Tongren Hospital Capital Medial University Beijing China; ^4^ Department of Neurointerventional Surgery Binzhou Medical University Hospital Binzhou China

**Keywords:** bioinformatics, inflammation and immune response, intracranial aneurysm, rupture, transcription factors

## Abstract

**Introduction:**

Genetic factors play a major part in mediating intracranial aneurysm (IA) rupture. However, research on the role of transcription factors (TFs) in IA rupture is rare.

**Aims:**

Bioinformatics analysis was performed to explore the TFs and related functional pathways involved in IA rupture.

**Results:**

A total of 63 differentially expressed transcription factors (DETFs) were obtained. Significantly enriched biological processes of these DETFs were related to regulation of myeloid leukocyte differentiation. The top 10 DETFs were screened based on the MCC algorithm from the protein–protein interaction network. After screening and validation, it was finally determined that CEBPB may be the hub gene for aneurysm rupture. The GSEA results of CEBPB were mainly associated with the inflammatory response, which was also verified by the experimental model of cellular inflammation in vitro.

**Conclusion:**

The inflammatory and immune response may be closely associated with aneurysm rupture. CEBPB may be the hub gene for aneurysm rupture and may have diagnostic value. Therefore, CEBPB may serve as the diagnostic signature for RIAs and a potential target for intervention.

## Graphical abstract

In this study, the differently expressed transcription factors between ruptured intracranial aneurysms and unruptured intracranial aneurysms and underlying functional mechanisms for aneurysm rupture were investigated by bioinformatics analysis. An amazing finding was that CEBPB may be the hub gene for ruptured intracranial aneurysms, which was closely associated with the inflammatory response and immune system process.

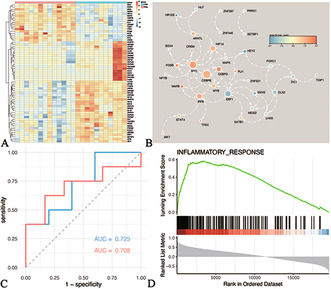

## 1 INTRODUCTION

Intracranial aneurysms (IAs) are abnormal pathological protrusion of the intracranial artery wall and occur in approximately 3.2% of the general population.^1^ The rupturing of an IA leads to subarachnoid hemorrhage, which results in a high rate of mortality and morbidity.^2^ The management of IA remains a challenge because most IAs are asymptomatic and have a low annual risk of rupture, while ruptured aneurysms lead to devastating results.^3^ Additionally, the current main treatment methods for IAs are invasive, which can lead to a variety of complications.^4^ Therefore, the risk of treatment needs to be weighed against the risk of rupture. Establishing a method to identify patients with IAs with a high risk of rupture to provide immediate intervention is critical.

Many studies have reported that genetic factors play a major part in mediating IA rupture, which has mainly focused on genes, microRNAs, long non‐coding RNAs, or circular RNAs.^5^, ^6^ Several studies performed gene expression profiling on the IA tissues with microarray data to acquire the key genes and pathways involved in IA rupture.^7^, ^8^, ^9^ However, the results of these studies might vary significantly because of the small samples, different data processing methods, and platform conditions. Transcription factors (TFs) are a class of proteins that bind to specific DNA sequences through their DNA‐binding domains, and are mainly involved in the regulation of gene expression.^10^ Exploring the TFs and related functional pathways involved in IA rupture may be valuable to elucidate the molecular mechanisms behind IA rupture. However, research on TFs that mediate IA rupture is scarce.

In this study, we performed bioinformatics analysis to obtain the differentially expressed TFs (DETFs) between unruptured IAs (UIAs) and ruptured IAs (RIAs). A protein–protein interaction (PPI) network was constructed to determine the potential hub genes, which were validated by other datasets.

## 2 MATERIALS AND METHODS

### 2.1 Data downloading and processing

Five microarray datasets were downloaded from the Gene Expression Omnibus database (www.ncbi.nlm.nih.gov/geo) for further analysis: GSE26969,^11^ GSE13353,^9^ GSE6551,^12^ GSE54083,^7^ and GSE15629.^8^ The gene symbols of GSE26969, GSE13353, and GSE6551 are based on the GPL570 platform. GSE54083 is based on the GPL4133 platform and GSE15629 is based on the GPL6244 platform. GSE26969 consists of three superficial temporal arteries (STA) and three UIA samples. GSE13353 includes 8 UIA and 11 RIA samples. GSE6551 contains five STA samples, three UIA samples, and two RIA samples. Ultimately, a gene expression profile dataset consisting of 11 UIA and 13 RIA samples based on the GPL 570 platform was selected from GSE26969, GSE13353, and GSE6551 for DETFs analysis. GSE54083 is comprised of 10 STA samples, 5 UIA samples, and 8 RIA samples. GSE15629 includes five middle meningeal artery samples, six UIA samples, and eight RIA samples. The gene expression data of UIA samples and RIA samples in the GSE54083 and GSE15629 datasets were used for validation.

### 2.2 Identification of DETFs

For the microarray data of GSE26969, GSE13353, and GSE6551 based on the GPL570 platform, batch effects were removed using the “ComBat” algorithm of “sva” R package (Supplemental Figure S1). Raw data were normalized and processed by the “limma” R package. Analysis of differentially expressed genes between UIAs and RIAs was performed. Genes with *p* value <0.05 and |log2 fold change (FC)| >1 were considered to be differentially expressed genes (DEGs).

Three TFs online datasets were downloaded: The Human Transcription Factors Database (Human TFs),^13^ Human Transcription Factor Database (Human TFDB),^14^ and Catalog of Inferred Sequence Binding Preferences (CIS‐BP) Database.^15^ TFs from the three databases were intersected with DEGs to acquire DETFs.

### 2.3 Functional enrichment analysis

Gene Ontology (GO) term functional annotation and Kyoto Encyclopedia of Genes and Genomes (KEGG) pathway analyses of the DETFs were performed by the “clusterProfiler” R package. GO enrichment analysis was categorized according to the biological process (BP), cellular component (CC), and molecular function (MF). The significantly enriched GO terms and KEGG pathways with a criterion of adjusted *p*‐value <0.05 were selected.

### 2.4 PPI network construction and identification of hub genes

Interaction networks of DETFs were constructed using the STRING database version 10.0 (http://string‐db.org/). Interaction visualization was performed using Cytoscape software. The CytoHubba plug‐in in Cytoscape software was applied for the calculation of top 10 DETFs via Multiscale Curvature Classification (MCC) algorithm. The MCODE plug‐in in Cytoscape software was applied for the sub‐network construction. Receiver operating characteristic (ROC) curve was applied to assess the diagnostic value of the potential TFs in discriminating RIAs from UIAs by the datasets GSE54083 and GSE15629. The hub genes were identified by the score on the scale‐free network and potential diagnostic value. Gene Set Enrichment Analysis (GSEA) for the hub genes was performed using the gene list ranked by Pearson correlation coefficient.

### 2.5 Cell culture

Human umbilical vein endothelial cells (HUVECs) were purchased from American Type Culture Collection (Rockville, MD) and were cultured in endothelial cell medium (ScienCell, USA) containing 5% fetal bovine serum, 1% endothelial cell growth additive, and 1% penicillin/streptomycin solution. Cell was cultured at 37°C in a humidified incubator of 5% CO_2_.

### 2.6 Cell transfection

For RNA interference, cells were spread in six‐well plates, and the cell density reached 70% after 24 h. Then, cells were transfected with siRNAs using Lipofectamine 2000 (Invitrogen), in accordance with the manufacturer's protocol. Three individual CEBPB siRNAs and scrambled negative control siRNA (si‐NC) were purchased from Invitrogen. At 48 h post‐transfection, the HUVECs were treated with lipopolysaccharide (LPS) (1 ug/mL, Sigma Aldrich) for 24 h.

For plasmid transfection, CEBPB sequences were synthesized and subcloned into pCDNA3.1 vector (Invitrogen), and then transfected by Lipofectamine 3000 (Invitrogen) into HUVEC cells, and empty pCDNA3.1 vector was used as a control. Forty‐eight hours after transfection, HUVEC cells were stimulated with LPS (1 ug/mL, Sigma‐Aldrich) for 24 h.

### 2.7 RNA extraction and qRT‐PCR analysis

Total cell RNA was extracted with TRIzol reagent (Invitrogen) according to the instructions. Primers were designed and synthesized according to the gene sequence provided by NCBI gene. Primer sequences are shown in Table 1. RNA was reverse transcribed into cDNA for qRT‐PCR using a reverse transcription kit (Vazyme), and real‐time PCR analysis was performed using Power SYBR Green (Vazyme). Real‐time PCR reaction system was 10 μL: ddH2O 3.6 μL, template cDNA 1.0 μL, upstream primer 0.2 μL, downstream primer 0.2 μL, and SYBR 5.0 μL. Three wells were set for each sample. The reaction conditions were as follows: the first stage predenaturation 95°C, 30 s; the second stage cycle 40 times, 95°C for 10 s, and 60°C for 30 s; and the dissolution curves of the third stage were 95°C for 15 s, 60°C for 60 s, and 95°C for 15 s.

**TABLE 1:** Primer sequences of related genes.

Genes	Sequences
CEBPB	Forward:5’‐GGAGCCCGTCGGTAATTT‐3’
	Reverse:5’‐TCTGCATGTGCGGTTGG‐3’
IL‐6	Forward:5’‐CACGGCCTTCCCTACTTC‐3’
	Reverse:5’‐TTTCCACGATTTCCCAGA‐3’
GAPDH	Forward:5’‐GGGAGCCAAAAGGGTCAT‐3’
	Reverse:5’‐GAGTCCTTCCACGATACCAA‐3’

### 2.8 ELISA

IL‐6 levels in cell culture medium supernatants were measured using IL‐6 ELISA Kit (Neobioscience Technology Company).

### 2.9 Statistical analysis

The variables from the quantitative real‐time PCR and ELISA analysis were tested for the Gaussian distribution. The Shapiro–Wilk normality test was used to identify the Gaussian distribution. The independent Student's *t*‐test was used to determine significant differences between two groups and multigroup comparison was performed by one‐way ANOVA. All data processing and statistical analyses will be performed using SPSS V.22.0 (IBM Corp). Statistical significance is defined as *p*‐value <0.05.

## 3 RESULTS

### 3.1 Identification of DETFs

**FIGURE 1:**
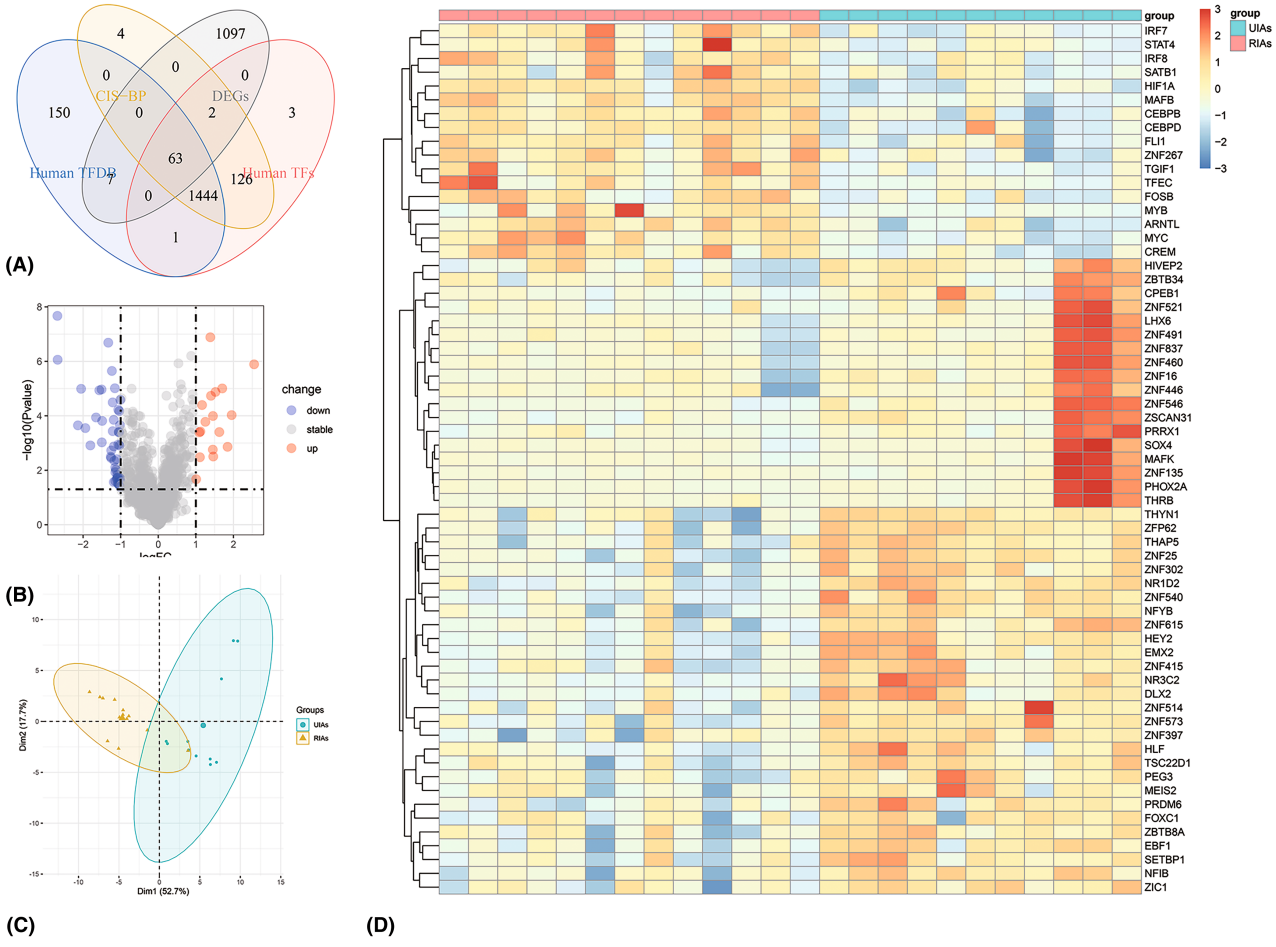
The differentially expressed transcription factors (DETFs) for ruptured intracranial aneurysm. (A) Identification of 63 DETFs from the intersection between the DEGs and 3 TFs databases (Human TFs, Human TFDB, and CIS‐BP). (B) Volcano plot for the presentation of 63 DETFs. Red dots represent the upregulated DETFs while blue dots represent downregulated DETFs. (C) Principal component analysis plot based on the 63 DETFs. (D) The heatmap of 63 DETFs between the ruptured intracranial aneurysms (RIAs) group and the unruptured intracranial aneurysms (UIAs) group.

According to the aforementioned criteria, a total of 1169 DEGs were acquired by R package “limma”, including 538 upregulated genes and 631 downregulated genes. The DEGs were intersected with 3 TF databases (The Human Transcription Factors, Human TFDB, and CIS‐BP), and 63 DETFs were obtained, including 17 upregulated TFs and 46 downregulated TFs (Figure 1A). The DETFs are presented in a volcano plot (Figure 1B), a principal component analysis plot (Figure 1C), and a heatmap (Figure 1D). All DETFs are listed in Table 2.

**TABLE 2:** The differentially expressed transcription factors (DETFs) (17 upregulated DETFs and 46 downregulated DETFs) between ruptured intracranial aneurysms and unruptured intracranial aneurysms.

DETFs	TF lists
Downregulated	NR1D2, ZNF540, HEY2, ZNF25, ZFP62, EMX2, NR3C2, NFYB, ZNF573, ZNF302, ZNF615, THAP5, NFIB, HLF, THYN1, ZNF397, EBF1, DLX2, TSC22D1, PRDM6, ZNF514, ZIC1, SETBP1, PEG3, ZNF546, ZSCAN31, CPEB1, HIVEP2, ZBTB8A, ZNF415, ZNF16, MEIS2, ZNF837, ZBTB34, ZNF460, FOXC1, PRRX1, PHOX2A, LHX6, ZNF491, ZNF446, SOX4, THRB, ZNF521, MAFK, ZNF135
Upregulated	HIF1A, MAFB, MYC, CREM, CEBPB, ARNTL, CEBPD, TGIF1, IRF7, MYB, FLI1, ZNF267, FOSB, IRF8, TFEC, STAT4, SATB1

### 3.2 Functional enrichment analysis of DETFs

**FIGURE 2:**
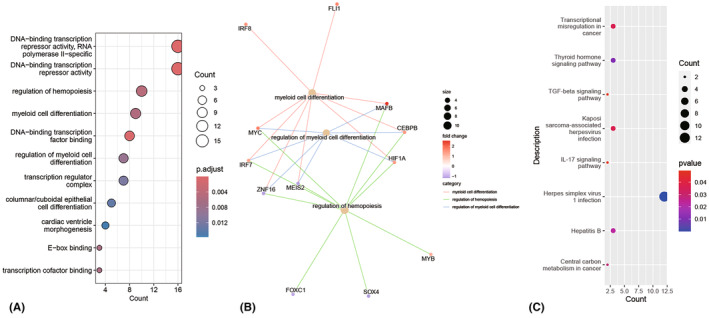
The Go terms and KEGG pathways enrichment analysis of 63 differentially expressed transcription factors for ruptured aneurysm. (A) Dot chart of the significantly enriched GO terms. (B) The presentation of top 3 significantly enriched GO terms including the transcription factors. (C) Dot chart of KEGG pathways colored by the p value.

Gene enrichment analysis on the DETFs was performed. With the threshold of adjusted *p* value <0.05, the DETFs were significantly enriched in 45 GO terms and 1 KEGG pathway. GO enrichment analysis demonstrated that the DETFs were significantly involved in the regulation of hemopoiesis, myeloid cell differentiation, regulation of leukocyte differentiation, transcription regulator complex, and DNA‐binding transcription repressor activity (Figure 2). The KEGG results showed that these DETFs were mainly involved in the herpes simplex virus 1 infection pathway. The significant GO terms and KEGG pathways are listed in Table 3.

**TABLE 3:** Significantly enriched GO terms and KEGG pathways for ruptured intracranial aneurysm.

ID	Category	Description	Count	Adjust *p*‐value
GO:1903706	BP	Regulation of hemopoiesis	10	0.00458
GO:0030099	BP	Myeloid cell differentiation	9	0.00543
GO:0045637	BP	Regulation of myeloid cell differentiation	7	0.00857
GO:0002065	BP	Columnar/cuboidal epithelial cell differentiation	5	0.0136
GO:0003208	BP	Cardiac ventricle morphogenesis	4	0.0157
GO:0048568	BP	Embryonic organ development	8	0.0157
GO:0007389	BP	Pattern specification process	8	0.0157
GO:0048844	BP	Artery morphogenesis	4	0.0157
GO:0010464	BP	Regulation of mesenchymal cell proliferation	3	0.0229
GO:0110111	BP	Negative regulation of animal organ Morphogenesis	3	0.0231
GO:0030522	BP	Intracellular receptor signaling pathway	6	0.0232
GO:0060840	BP	Artery development	4	0.0318
GO:1902105	BP	Regulation of leukocyte differentiation	6	0.0318
GO:0021953	BP	Central nervous system neuron differentiation	5	0.0326
GO:0010463	BP	Mesenchymal cell proliferation	3	0.0326
GO:0048839	BP	Inner ear development	5	0.0334
GO:1903708	BP	Positive regulation of hemopoiesis	5	0.0369
GO:0021892	BP	Cerebral cortex GABAergic interneuron Differentiation	2	0.0375
GO:0045646	BP	Regulation of erythrocyte differentiation	3	0.0375
GO:0072132	BP	Mesenchyme morphogenesis	3	0.0399
GO:0002761	BP	Regulation of myeloid leukocyte differentiation	4	0.0399
GO:0043583	BP	Ear development	5	0.0424
GO:0060563	BP	Neuroepithelial cell differentiation	3	0.0424
GO:0003206	BP	Cardiac chamber morphogenesis	4	0.0424
GO:0003231	BP	Cardiac ventricle development	4	0.0424
GO:0097154	BP	GABAergic neuron differentiation	2	0.0449
GO:0005667	CC	Transcription regulator complex	7	0.0117
GO:0001227	MF	DNA‐binding transcription repressor activity, RNA polymerase II‐specific	16	4.13E‐13
GO:0001217	MF	DNA‐binding transcription repressor activity	16	4.13E‐13
GO:0140297	MF	DNA‐binding transcription factor binding	8	0.000405
GO:0070888	MF	E‐box binding	3	0.00598
GO:0001221	MF	Transcription cofactor binding	3	0.00598
GO:0004879	MF	Nuclear receptor activity	3	0.00598
GO:0098531	MF	Ligand‐activated transcription factor activity	3	0.00598
GO:0000900	MF	Translation repressor activity, mRNA regulatory element binding	2	0.00948
GO:0070491	MF	Repressing transcription factor binding	3	0.0120
GO:0061629	MF	RNA polymerase II‐specific DNA‐binding Transcription factor binding	5	0.0120
GO:0033613	MF	Activating transcription factor binding	3	0.0132
GO:0030371	MF	Translation repressor activity	2	0.0167
GO:0035035	MF	Histone acetyltransferase binding	2	0.0178
GO:0001223	MF	Transcription coactivator binding	2	0.0178
GO:0042826	MF	Histone deacetylase binding	3	0.0268
GO:0051879	MF	Hsp90 protein binding	2	0.0321
GO:0001046	MF	Core promoter sequence‐specific DNA binding	2	0.0355
GO:0001102	MF	RNA polymerase II‐activating transcription factor binding	2	0.0355
hsa05168	KEGG	Herpes simplex virus 1 infection	12	7.95e‐06

Abbreviations: BP, biological process; CC, cellular component; GO, Gene Ontology; KEGG, Kyoto Encyclopedia of Genes and Genomes; MF, molecular function.

### 3.3 PPI network construction and hub gene identification

**FIGURE 3:**
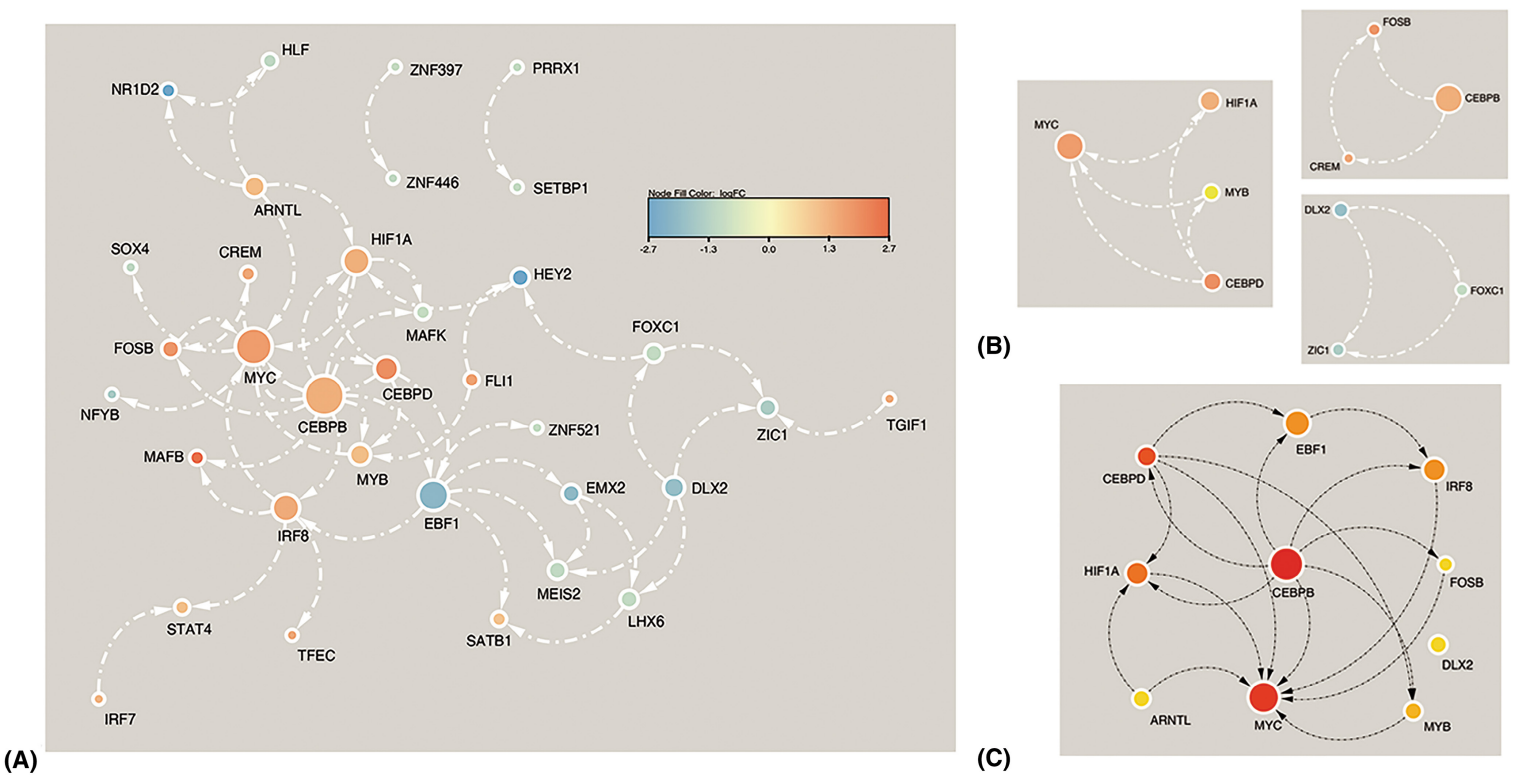
Protein–protein interaction (PPI) network construction and analysis. (A) PPI network of the 63 differentially expressed transcription factors (DETFs) was constructed. (B) Three subnetworks were obtained by the MCODE plug‐in in Cytoscape software. (C) The top 10 DETFs were screened by the MCC algorithm.

**FIGURE 4:**
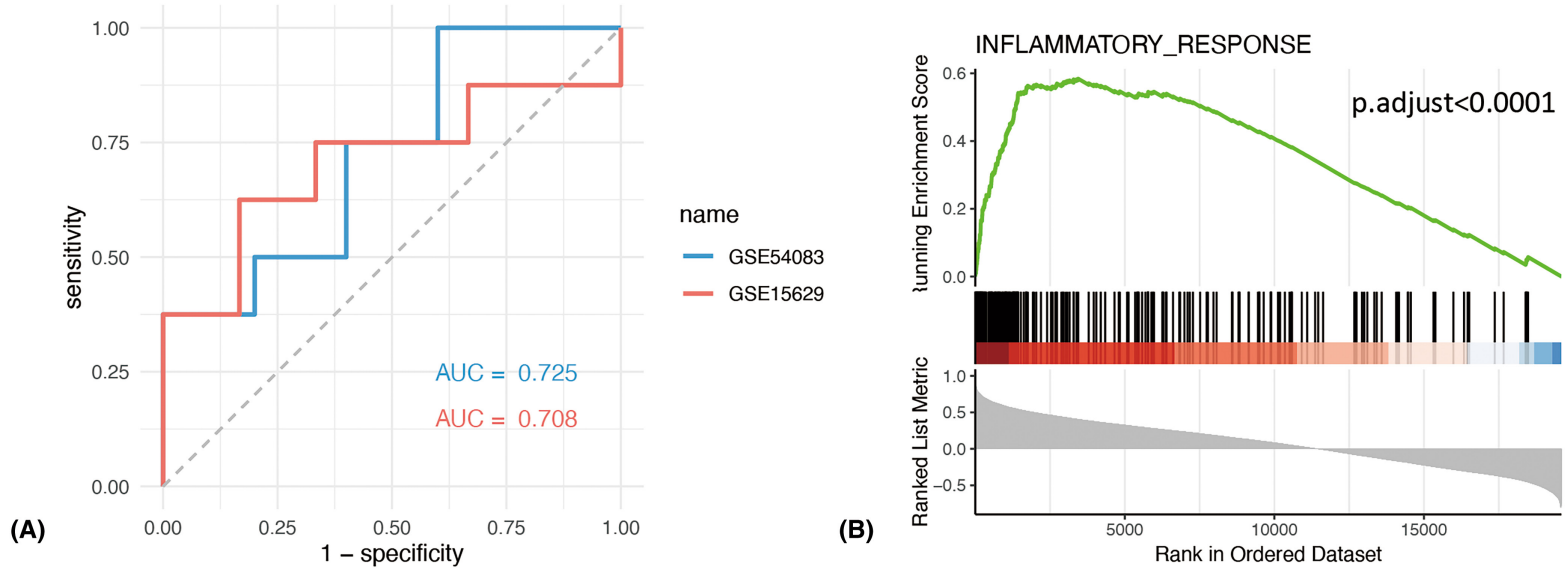
Validation of the hub gene (CEBPB). (A) the ROC curve of CEBPB in the datasets of GSE54083 and GSE15629. (B) The results of GSEA analysis showed that CEBPB was mainly associated with inflammatory response.

A PPI network of DETFs was constructed using STRING online database, and the PPI network was visualized by Cytoscape software. The PPI network of the 63 DETFs included 34 nodes and 51 edges (Figure 3A). Three subnetworks (MYB‐MYC‐CEBPD‐HIF1A, CEBPB‐FOSB‐CREM, and DLX2‐FOXC1‐ZIC1) were obtained by the MCODE plug‐in in Cytoscape software (Figure 3B). The top 10 DETFs were screened using the MCC algorithm with the cytoHubba plug‐in of Cytoscape software (Table 4). The connection network between the top 10 DETFs is shown in Figure 3C. With the filtered MCC score >20, two potential hub genes (CEBPB and MYC) were selected. ROC curve was applied to assess the diagnostic value of CEBPB and MYC using GSE54083 and GSE15629 datasets. The area under the curve (AUC) values of CEBPB for GSE54083 and GSE15629 were 0.725 and 0.708, respectively (Figure 4A); while the AUC values of MYC for GSE54083 and GSE15629 were 0.525 and 0.521, respectively. These results indicated that CEBPB had diagnostic value for aneurysm rupture and that CEBPB may be the hub gene for RIAs. The GSEA results of CEBPB were mainly associated with inflammatory response (Figure 4B).

**TABLE 4:** Top 10 differentially expressed transcription factors in PPI network ranked by MCC method.

Gene symbol	Official full name	Score
CEBPB	CCAAT Enhancer‐Binding Protein Beta	26
MYC	MYC Proto‐Oncogene, BHLH Transcription Factor	20
CEBPD	CCAAT Enhancer‐Binding Protein Delta	14
HIF1A	Hypoxia‐Inducible Factor 1 Subunit Alpha	11
EBF1	EBF Transcription Factor 1	8
IRF8	Interferon Regulatory Factor 8	8
MYB	MYB Proto‐Oncogene, Transcription Factor	7
DLX2	Distal‐Less Homeobox 2	4
FOSB	FosB Proto‐Oncogene, AP‐1 Transcription Factor Subunit	4
ARNTL	Aryl Hydrocarbon Receptor Nuclear Translocator Like	4

### 3.4 Experimental verification

**FIGURE 5:**
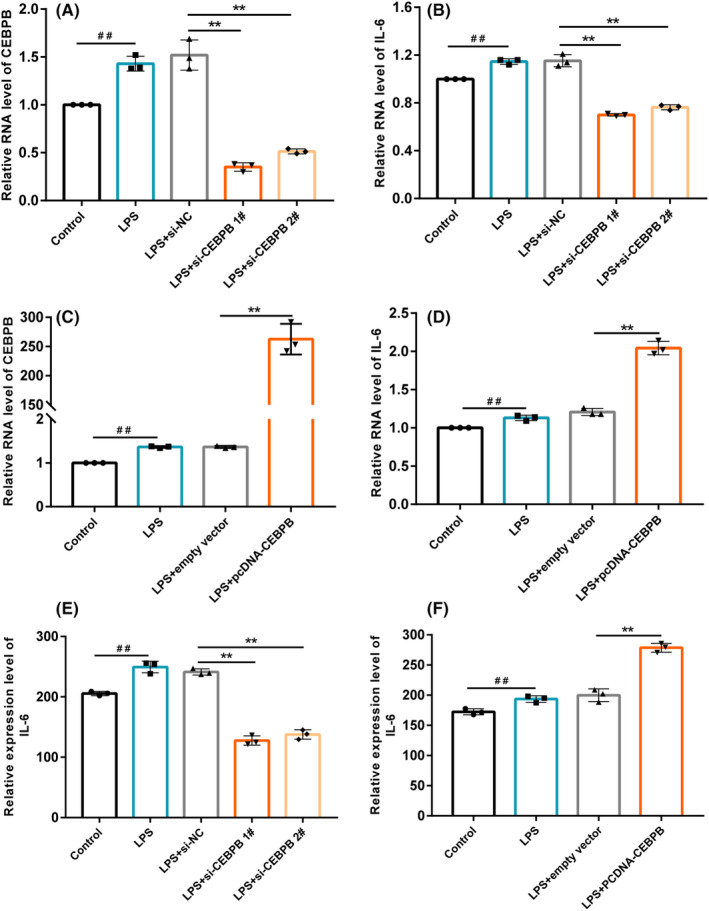
Experimental verification. (A‐D): qRT‐PCR was used to detect the expression of CEBPB and IL‐6 RNA. (E, F) The expression level of IL‐6 in the supernatant was detected by ELISA. si‐NC, negative control siRNA; si‐CEBPB, CEBPB siRNA; empty vector, negative control pcDNA‐CEBPB; pcDNA‐CEBPB, CEBPB overexpression plasmid. ##*p* < 0.01 compared with the control group;**p < 0.01 compared with the LPS + si‐NC group or LPS + empty vector group.

To further verify whether CEBPB is associated with inflammatory immune response, we stimulated HUVECs with LPS to establish an experimental model of cellular inflammation in vitro. CEBPB siRNA was constructed to knock down the expression of CEBPB in HUVEC cells, and its knockdown efficiency was detected by qRT‐PCR. The results showed that LPS could stimulate the upregulation of CEBPB, and siRNA could significantly knock down the expression of CEBPB (Figure 5A). Previous studies have shown that CEBPB, also named NF‐IL6, can bind to the promoter of IL‐6 and promote the expression of IL‐6. We further detected the mRNA expression of IL‐6 in HUVEC cells with CEBPB knockdown, and qRT‐PCR results showed that the expression of IL‐6 was upregulated under LPS stimulation, while the expression of IL‐6 in CEBPB knockdown group was decreased (Figure 5B). Then, we constructed CEBPB overexpression plasmid, and the expression efficiency after transfection into HUVEC cells is shown in Figure 5C. The expression of IL‐6 mRNA was detected by qRT‐PCR, and the results showed that the expression of IL‐6 was significantly increased in CEBPB overexpression group compared with the control group (Figure 5D). At the same time, ELISA was used to detect the expression of IL‐6 in the medium supernatant, and the results showed that compared with the control group, the expression of IL‐6 in the CEBPB knockdown group was significantly decreased, while the expression of IL‐6 in the overexpression group was significantly increased (Figure 5E,F). These results indicate that CEBPB can upregulate the expression of IL‐6 in vascular endothelial cells, thereby affecting the inflammatory response.

## 4 DISCUSSION

Establishing methods to distinguish IAs with high rupture risk from IAs with low rupture risk is critical to be able to provide timely intervention for patients with high aneurysm rupture risk. Many studies have shown that genetic factors play a major part in aneurysm rupture.^5^, ^6^ Therefore, in this study, the DETFs between RIAs and UIAs and underlying functional mechanisms for aneurysm rupture were investigated by bioinformatics analysis. Our analysis identified 63 DETFs. PPI network analysis and validation by two datasets revealed that CEBPB may be the hub gene for RIAs. Functional enrichment analysis showed that these DETFs and the hub gene (CEBPB) were closely associated with the inflammatory response and immune system process. The results of in vitro experimental verification indicated that the hub gene (CEBPB) could upregulate the expression of IL‐6 in vascular endothelial cells, thereby affecting the inflammatory response.

Inflammatory infiltration has been shown to participate in the process of aneurysm rupture.^7^, ^16^ Inflammatory cell infiltrations and inflammatory mediators in the aneurysmal wall may result in critical weakening of the aneurysmal wall and ultimate aneurysm rupture.^17^ Moreover, cytokines and extracellular matrix‐degrading proteolytic enzymes secreted from inflammatory cells promote fragmentation of aneurysmal wall structures and induce cell death.^18^ In this study, the results of GO enrichment analysis indicated that the DETFs were closely associated with the regulation of myeloid leukocyte differentiation. Single‐gene GSEA showed that the hub gene (CEBPB) was mainly enriched for gene sets associated with the inflammation response. The in vitro experimental verification also indicated that CEBPB can upregulate the expression of IL‐6 in vascular endothelial cells, thereby affecting the inflammatory response. These results of the study confirmed that inflammatory and immune responses may be significantly involved in the aneurysm rupture, similar to the findings of previous studies.

The KEGG pathways for these 63 DETFs were majorly enriched in the herpes simplex virus 1 infection pathway. Herpes simplex virus 1 is a common human pathogen that infects orofacial mucosal surfaces at the beginning and subsequently infiltrates the nervous system. It could undergo dormancy in sensory neurons, evades its host immune responses, and can be reactivated from the latent state. Upon reactivation, it can infect arteries, resulting in an inflammatory process in the vessel wall.^19^, ^20^ Matrix metalloproteinase‐9 (MMP‐9) may be a key factor in this process. MMP‐9 activity leads to damage to the cerebral vasculature and degradation of the neurovascular matrix.^21^, ^22^

The PPI network analysis and validation by two datasets indicate that CEBPB may be the hub gene for RIAs, with diagnostic values. CEBPB is an important transcription factor that could mediate the gene expression involved in inflammatory and immune responses. CEBPB binds the regulatory elements of cytokines genes and plays a part in the regulation of inflammation, which is essential for gene expression involved in activated macrophages, and plays an important role in immune responses such as the CD4(+) T‐cell response. In this study, single‐gene GSEA for CEBPB was mainly enriched in the inflammation response, which demonstrated that inflammation response was significantly involved in the pathophysiology of aneurysm rupture.

This study has several limitations. Because of the difficulty in acquiring materials from aneurysms and artery tissues, the sample size was limited. Further studies with a larger sample size are required to identify the results. Additional research is necessary to investigate the detailed molecular mechanisms and biological functions of the hub gene involved in the aneurysm rupture.

## 5 CONCLUSION

Inflammatory and immune responses may be closely associated with aneurysm rupture. CEBPB may be the hub gene for RIAs, and have diagnostic value. Therefore, CEBPB may serve as a diagnostic signature for aneurysm rupture and a potential target for intervention. More research is needed to verify the results.

## AUTHOR CONTRIBUTIONS

Zhongbin Tian and Xuefang Wu performed the analysis and manuscript writing. Zhongbin Tian and Baorui Zhang acquired and processed the data. Xuefang Wu performed the experimental verification. Chao Wang and Wei Li designed the research.

## FUNDING INFORMATION

This work was supported by the Chinese Society of Clinical Oncology Foundation (Y‐HR2017‐035 and Y‐sy2018‐242) and the Natural Science Foundation of Shandong Province (ZR2023MH001).

## CONFLICT OF INTEREST STATEMENT

The authors declare that they have no competing interests.

## Supporting information


Figure S1.

## Data Availability

The data analyzed in this study was available from the Gene Expression Omnibus database (www.ncbi.nlm.nih.gov/geo). The authors agree to share any data on request. Any data from this study are available by contacting the corresponding author.
