# Supplementary figures and images for: Transcription factor CEBPB mediates intracranial aneurysm rupture by inflammatory and immune response
Source: CNS Neurosci Ther. 2024 Feb 8;30(2):e14603. doi: 10.1111/cns.14603 (PMC10853640; doi:10.1111/cns.14603)

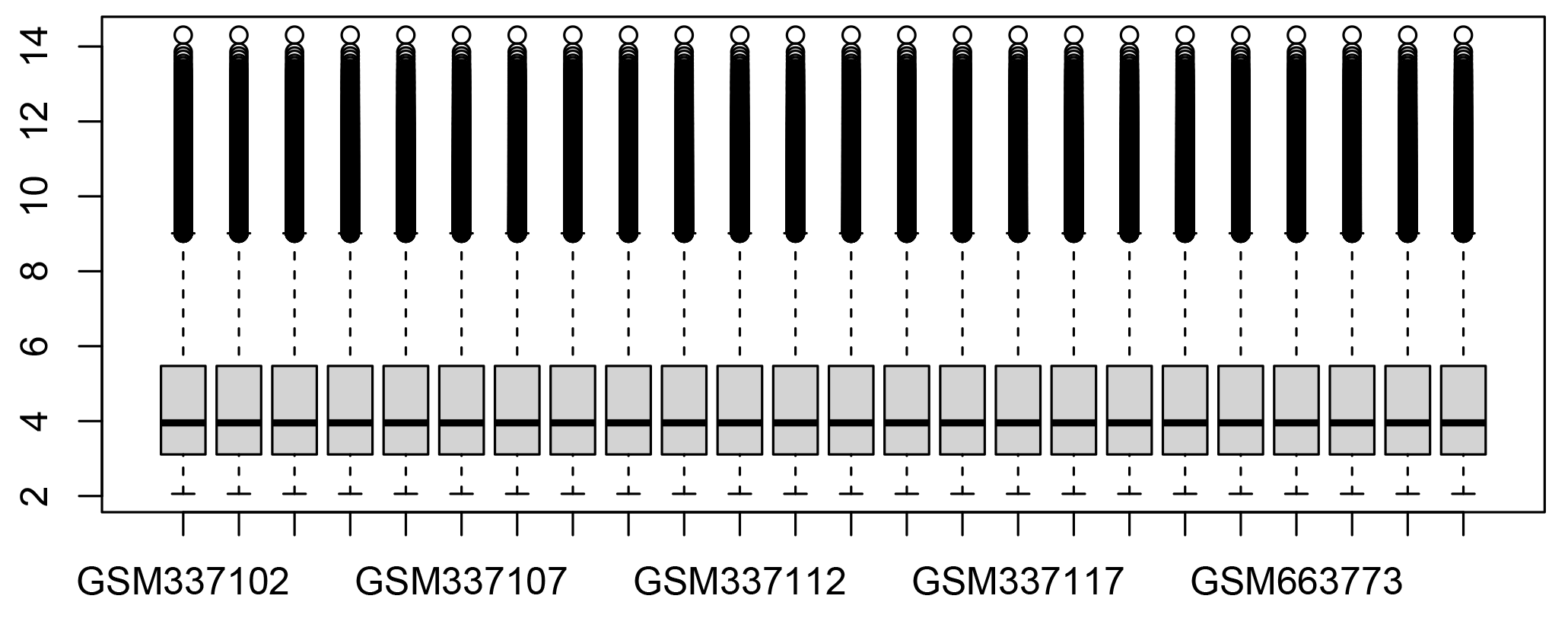

Supplement: Supplementary file 1 — Figure S1. [file CNS-30-e14603-s001.tif]
